# Oxophilic Yb_2_O_3_ nanoparticles as radical scavengers to enhance the durability of Fe–N–C oxygen reduction catalysts

**DOI:** 10.1002/smo2.70049

**Published:** 2026-05-06

**Authors:** Jiayu Liu, Shuang Li, Huijuan Li, Xin Wan, Jianglan Shui

**Affiliations:** ^1^ School of Materials Science and Engineering Beihang University Beijing China; ^2^ Tianmushan Laboratory Hangzhou China

**Keywords:** Fe–N–C, fuel cells, lanthanide sesquioxides, oxophilicity, radical scavengers

## Abstract

The practical application of Fe–N–C catalysts in proton exchange membrane fuel cells is substantially limited by their susceptibility to radical‐induced degradation of the active Fe–N_4_ sites, which severely compromises long‐term durability. To address this issue, we report a strategy of immobilizing Yb_2_O_3_ nanoparticles as efficient radical scavengers adjacent to Fe–N_4_ sites. The remarkable scavenging capability of Yb_2_O_3_, surpassing that of conventional cerium oxide, is attributed to its favorable oxophilicity, which facilitates radical capture and conversion. The resulting Fe–NC/Yb_2_O_3_ catalyst exhibits outstanding oxygen reduction reaction performance, featuring an initial half‐wave potential of 0.809 V and a negligible decay of only 19 mV after 10,000 cycles. In H_2_–air fuel cell tests, it retains 87.1% of its initial current density at 0.5 V over 20 h. Theoretical calculations indicate that the performance enhancement stems from strengthened radical adsorption and an optimized *d*‐band center of the Fe–N_4_ sites induced by Yb_2_O_3_. This work pioneers the use of a lanthanide sesquioxide for fuel cell catalyst protection, offering an effective strategy to overcome the activity–stability trade‐off in non‐precious metal catalysts.

## Graphical abstract

Fe–N–C catalysts suffer from radical attack during oxygen reduction. By immobilizing oxophilic Yb_2_O_3_ nanoparticles near Fe–N_4_ sites as efficient radical scavengers, the catalyst achieves enhanced activity and stability.

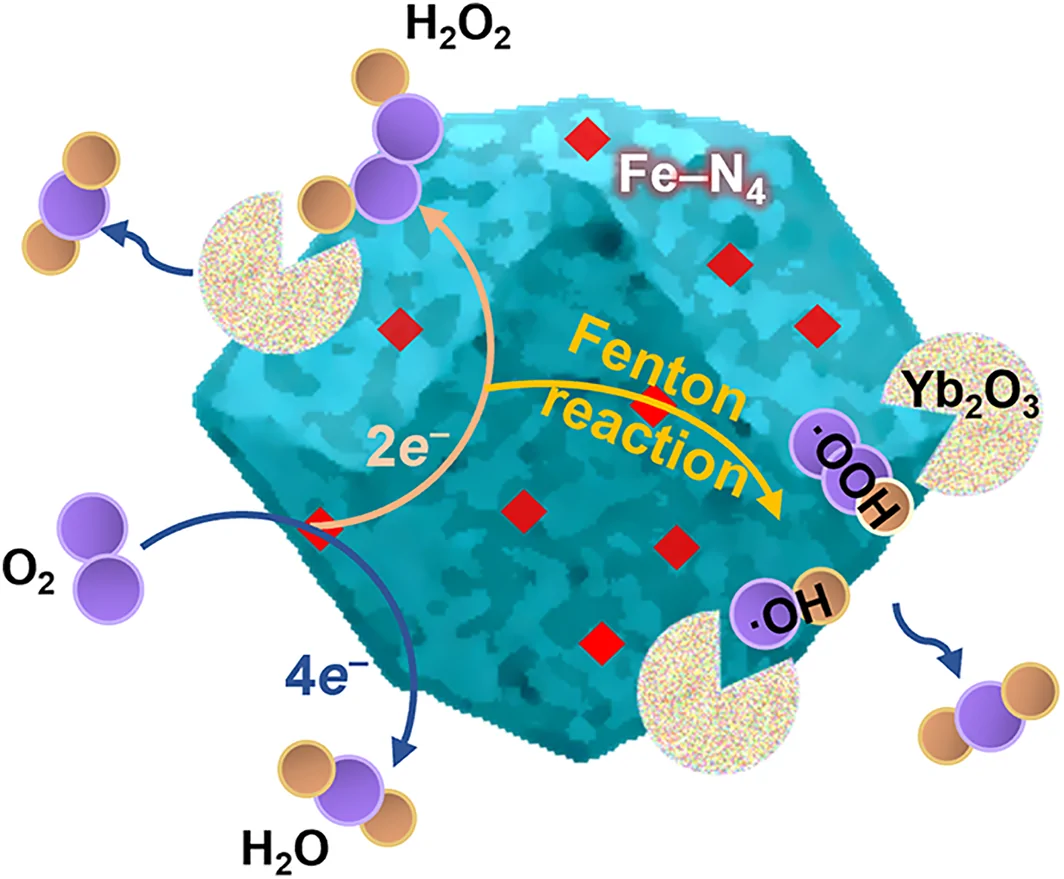

## 1 INTRODUCTION

Proton exchange membrane fuel cells (PEMFCs) are a key technology for decarbonizing transportation owing to their zero emissions and high energy efficiency. However, their widespread adoption is limited by the cost and durability issues of cathode catalysts for the oxygen reduction reaction (ORR). Although platinum‐based catalysts exhibit excellent activity and stability in acidic environments, their high cost and scarcity pose significant barriers.[^1^, ^2^, ^3^, ^4^] Carbon‐supported, nitrogen‐coordinated transition metal single‐atom catalysts (M–N–C, where M = Fe, Co, etc.) have thus emerged as promising non‐precious alternatives to expensive Pt catalysts for ORR in PEMFCs due to their excellent performance.[^5^, ^6^] Among them, Fe–N–C catalysts are considered the most viable, with performance approaching that of Pt‐based materials.[^7^, ^8^, ^9^, ^10^, ^11^]

The remarkable performance of Fe–N–C catalysts is largely attributed to the construction of high‐density, atomically dispersed Fe–N_4_ active sites, which confer high intrinsic ORR activity.[^12^, ^13^, ^14^] However, high iron content exacerbates the Fenton effect, creating a trade‐off between activity and stability. Under the harsh operating conditions of PEMFC cathodes, including strong acidity, high potential, and highly oxidizing atmosphere, these dense Fe–N_4_ sites can trigger a self‐reinforcing degradation process.[^15^, ^16^, ^17^, ^18^, ^19^, ^20^, ^21^, ^22^] Reaction byproducts such as H_2_O_2_ generate reactive oxygen radicals (e.g., ·OH, HO_2_·) via the Fenton reaction, which readily attack the carbon matrix and Fe–N_4_ sites, leading to iron leaching and structural collapse. The degraded structure, in turn, produces more H_2_O_2_, while leached iron ions further catalyze radical generation, intensifying the degradation cycle. Consequently, the operational lifetime of most Fe–N–C catalysts remains limited to less than 500 h, far below the 8000 h required for commercial applications.[^23^, ^24^, ^25^, ^26^] Resolving this stability issue is therefore essential for the practical application of Fe–N–C catalysts.

Incorporating radical scavengers offers a promising route to mitigate radical‐induced degradation.[^27^, ^28^, ^29^, ^30^, ^31^] Cerium oxide (CeO_2_) has been widely studied in this regard due to its reversible Ce^3+^/Ce^4+^ redox pair.[^32^, ^33^, ^34^, ^35^, ^36^] However, its effectiveness under operational conditions is often compromised by Ce dissolution. The dissolved Ce^4+^, a strong oxidant, can further lead to ionomer contamination and corrosion of Pt or carbon supports, thereby limiting practical application.[^37^, ^38^] Recently, Ta–TiO_*x*_ has been reported as a potential alternative, outperforming conventional CeO_2_.^[39]^ However, the Ta–TiO_*x*_ nanoparticles were supported on carbon black and only physically mixed with Fe–N–C catalysts, leaving too much distance between the scavenger and active sites. This likely allows radical damage to occur during migration. Thus, improving both the structural stability and spatial arrangement of scavengers is necessary to enhance their efficacy.

In this work, we designed a scavenger with optimized spatial positioning and electronic properties for more effective protection. We selected bixbyite‐type ytterbium oxide (Yb_2_O_3_) for its high thermodynamic stability and favorable oxophilicity. Among lanthanide sesquioxides, Yb_2_O_3_ possesses optimal OH‐binding energy (neither too strong nor too weak).^[40]^ This well‐balanced property promotes efficient radical adsorption followed by rapid catalytic conversion, which is crucial for high scavenging activity. Despite these advantageous properties, its application in PEMFC catalysts has rarely been explored. Here, Yb_2_O_3_ nanoparticles were anchored near Fe–N_4_ sites in an Fe–N–C matrix (denoted as Fe–NC/Yb_2_O_3_) via a two‐step doping process. This spatial design enables the rapid scavenging of detrimental radicals produced during ORR before they can degrade the active sites. The resulting catalyst exhibits significantly improved activity and durability compared to conventional Fe–N–C and Fe–NC/CeO_2_. This work provides an effective radical scavenging platform to extend the lifetime of non‐precious metal catalysts in fuel cells and could be adapted to other systems that must cope with detrimental oxygen radicals.

## 2 RESULTS AND DISCUSSION

### 2.1 Catalyst synthesis and characterizations

The Fe–NC/Yb_2_O_3_ composite catalyst was prepared via an impregnation method. Its key structural feature is the spatial proximity between high‐density Fe–N_4_ active sites and Yb_2_O_3_ radical scavengers. Using 1,10‐phenanthroline (Phen)‐coated zeolitic imidazolate framework‐8 (ZIF‐8) as a precursor, we obtained a nitrogen‐doped carbon (NC) substrate after high‐temperature calcination. This NC substrate retained well‐defined dodecahedral frameworks with a size of approximately 300 nm (Figure S1) and exhibited a high proportion of pyridinic nitrogen. The latter likely originates from the conversion of phenanthroline into a pyridinic‐N‐rich carbon shell (Figure S2), which is favorable for anchoring Fe atoms.^[41]^ Yb was introduced by impregnating Yb(NO_3_)_3_·5H_2_O, followed by annealing, which yielded a uniform dispersion of Yb_2_O_3_ nanoparticles (∼10 nm) on the NC support (Figure S3). Fe was subsequently doped through sublimed ferrocene vapor at 120°C, which was anchored by pyridinic‐N‐rich structures on the surface, forming high‐density Fe–N_4_ sites. By systematically tuning the mass ratio of the NC substrate to the Yb(NO_3_)_3_·5H_2_O precursor, we identified an optimal NC: Yb(NO_3_)_3_·5H_2_O ratio of 4:1, which balanced ORR activity and long‐term stability (Figure S4). Inductively coupled plasma optical emission spectroscopy analysis determined the Yb content in the Fe–NC/Yb_2_O_3_ composite to be 9.8 wt%, corresponding to a Yb_2_O_3_ loading of 11.6 wt%. A conventional Fe–NC/CeO_2_ composite catalyst was synthesized as a control sample following the same procedure used for Fe–NC/Yb_2_O_3_, except that Yb(NO_3_)_3_·5H_2_O was replaced with an equal mass of Ce(NO_3_)_3_·6H_2_O. Inductively coupled plasma optical emission spectroscopy analysis revealed a Ce content of 7.9 wt% in Fe–NC/CeO_2_, corresponding to a CeO_2_ loading of 9.7 wt%.

Transmission electron microscopy (TEM) confirmed the uniform dispersion of Yb_2_O_3_ nanoparticles on the catalyst (Figure 1a). A lattice spacing of 0.301 nm, measured from the high‐resolution TEM (HR‐TEM) image, matches the (222) plane (JCPDS No. 88–2161) of cubic Yb_2_O_3_ (Figure 1b). X‐ray diffraction confirmed the cubic crystal structure of Yb_2_O_3_ (Figure 1c). Furthermore, high‐angle annular dark‐field scanning TEM (HAADF‐STEM) imaging and corresponding elemental mapping revealed the homogeneous distribution of atomic Fe across the NC substrate (Figure 1d). The Yb signal also exhibited a relatively even distribution, attributable to the small size and high density of the Yb_2_O_3_ nanoparticles.

**FIGURE 1:**
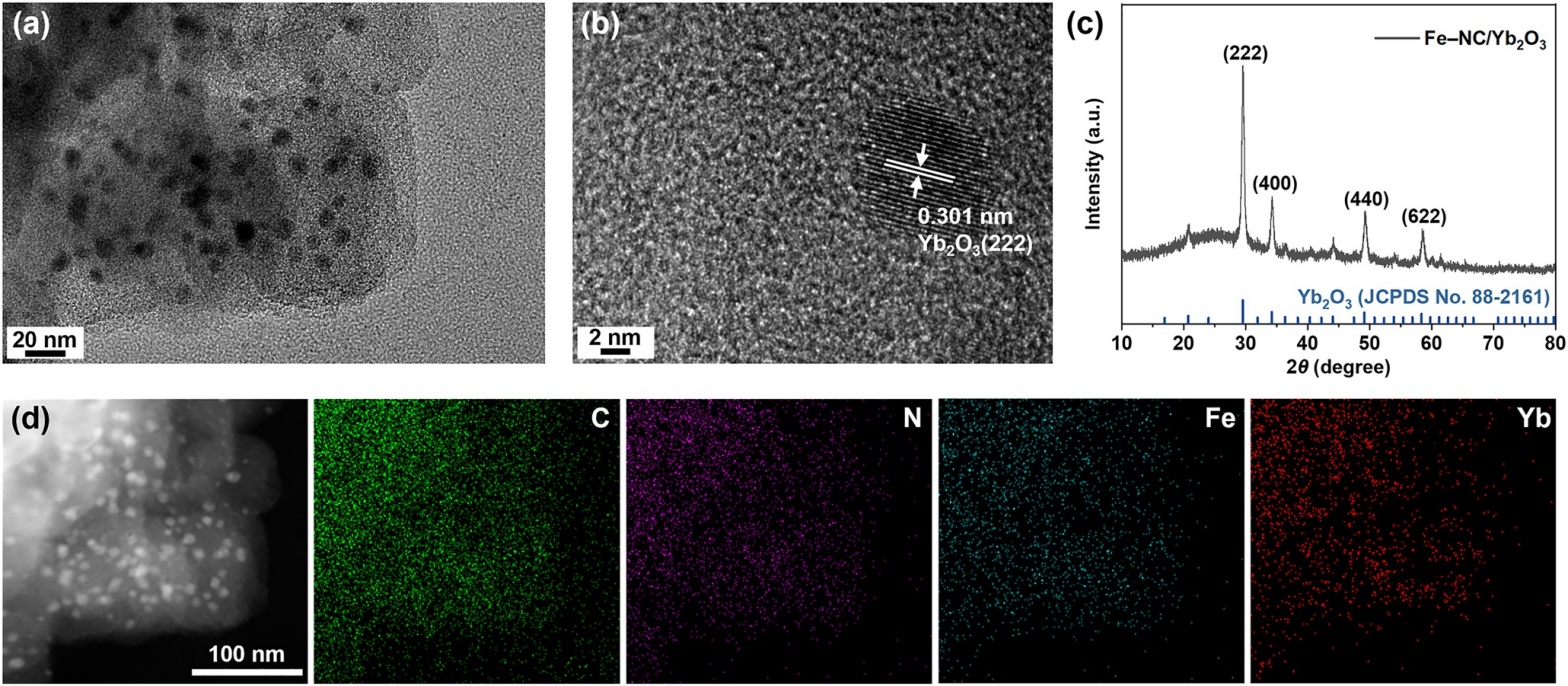
Structural characterizations of Fe–NC/Yb_2_O_3_. (a) TEM image. (b) HR‐TEM image. (c) XRD pattern. (d) HAADF‐STEM image and corresponding elemental mapping. TEM, Transmission electron microscopy; XRD, X‐ray diffraction.

X‐ray photoelectron spectroscopy N 1s spectra of both Fe–NC/Yb_2_O_3_ and Fe–N–C revealed typical nitrogen species, including pyridinic‐N (397.8 eV), pyrrolic‐N (399.2 eV), graphitic‐N (400.7 eV), and oxidized N (403.1 eV) (Figure S5). Consistent with the earlier observation, a high proportion of pyridinic nitrogen was present again attributed to the Phen‐derived pyridinic‐N‐rich carbon shell. The Fe content remained relatively low, resulting in weak signals in the Fe 2*p* spectrum (Figure S6). The Yb 4d X‐ray photoelectron spectroscopy spectrum (Figure S7) displayed five peaks consistent with the Yb^3+^ oxidation state.

### 2.2 Radical scavenging activity

To evaluate the radical scavenging activity of Fe–N–C, Fe–NC/CeO_2_, and Fe–NC/Yb_2_O_3_ catalysts, we employed UV‐Vis spectroscopy to monitor the change in radical concentration using a 2,2‐azinobis(3‐ethylbenzothiazoline‐6‐sulfonate) (ABTS) radical elimination assay.^[42]^ This assay is widely adopted because ABTS can be oxidized by radicals, resulting in a characteristic increase in absorbance at 417 nm and a visual color change from colorless to green (Figure 2a). As shown in Figure 2b and Figure S8, both Fe–NC/CeO_2_ and Fe–NC/Yb_2_O_3_ effectively eliminated radicals, as indicated by the decrease in absorbance at 417 nm and the lightening of the solution color. We attribute this absorbance decay to the higher radical‐eliminating ability of the oxide scavengers (CeO_2_ or Yb_2_O_3_), which reduces the oxidation of the ABTS molecular probe. Notably, Fe–NC/Yb_2_O_3_ more significantly suppressed the formation of ABTS radicals compared to Fe–NC/CeO_2_. These results clearly indicate that Yb_2_O_3_, when integrated with the Fe–NC framework, exhibits superior radical scavenging efficiency over conventional CeO_2_ under the present acidic and oxidative conditions.

**FIGURE 2:**
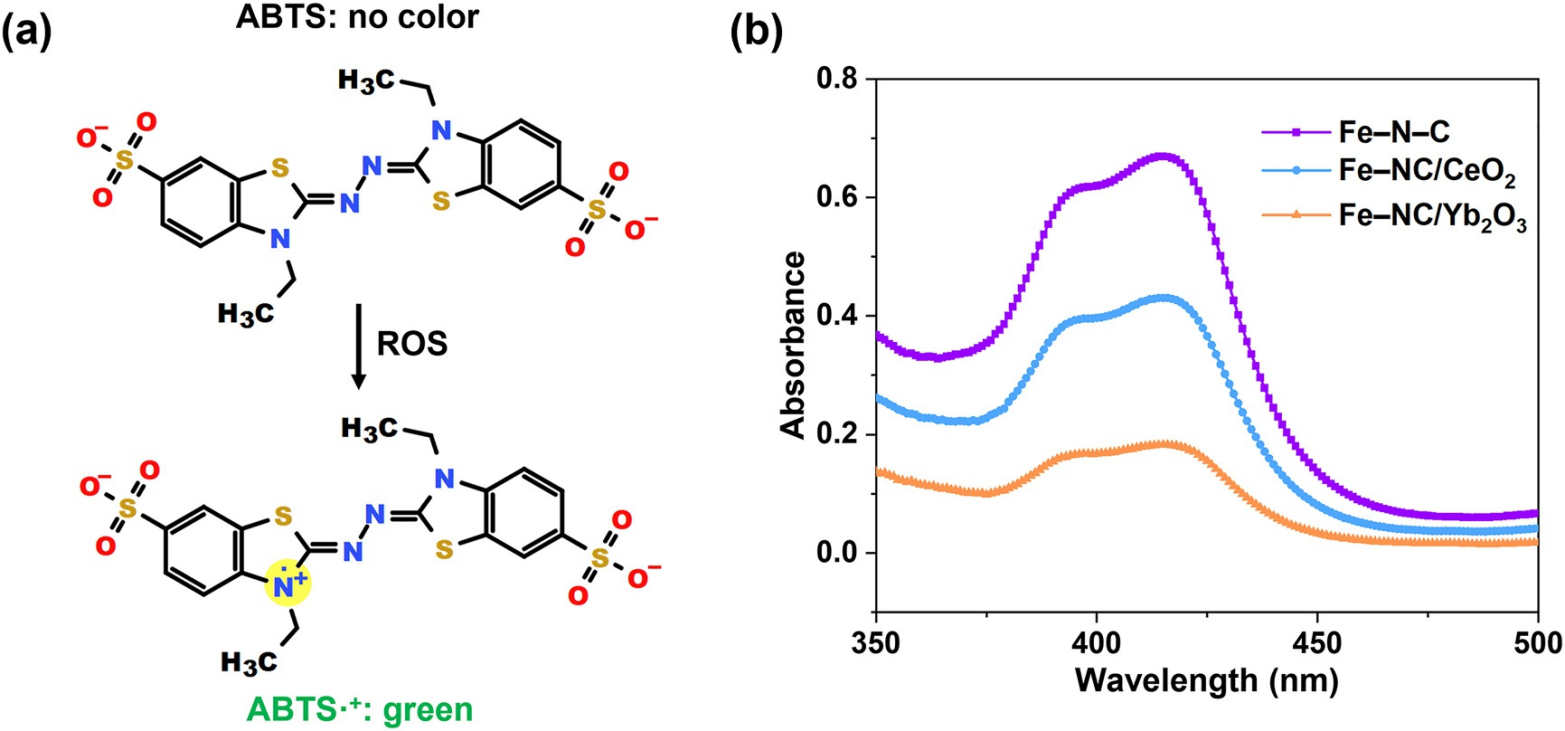
Radical scavenging activity. (a) Color reaction between ROS and ABTS. (b) UV‐vis absorption spectra of 0.1 м H_2_SO_4_ + H_2_O_2_ + ABTS solutions with the addition of Fe–N–C, Fe–NC/CeO_2_, or Fe–NC/Yb_2_O_3_ after 10 min reaction.

### 2.3 Electrocatalytic performance and stability

The ORR performance of the synthesized catalysts was evaluated using a rotating ring‐disk electrode in O_2_‐saturated 0.5 м H_2_SO_4_ solution. As shown in Figure 3a, Fe–NC/Yb_2_O_3_ exhibited the highest ORR activity, with a half‐wave potential (*E*_1/2_) of 0.809 V (vs. RHE), which was 11 and 24 mV higher than that of Fe–NC/CeO_2_ and Fe–N–C, respectively. Fe–NC/Yb_2_O_3_ also delivered the highest kinetic current density of 6.52 mA cm^−2^ at 0.8 V (Figure 3b) and showed quite a small Tafel slope among all catalysts (Figure 3c), indicating an accelerated rate‐determining step. Within the potential range of 0.2–0.8 V, all the catalysts exhibited H_2_O_2_ yields below 5% and electron transfer numbers above 3.9 (Figure S9). Among them, Fe–NC/Yb_2_O_3_ presented a slightly lower H_2_O_2_ yield and a higher electron transfer number, suggesting a more complete four‐electron ORR pathway. Moreover, the iron‐free NC/Yb_2_O_3_ sample showed negligible ORR activity similar to the metal‐free NC (Figure S10), confirming that Yb_2_O_3_ itself does not contribute significantly to the catalytic performance. Therefore, Yb_2_O_3_ is not ORR active but rather improves the intrinsic activity of the adjacent Fe–N_4_ sites.

**FIGURE 3:**
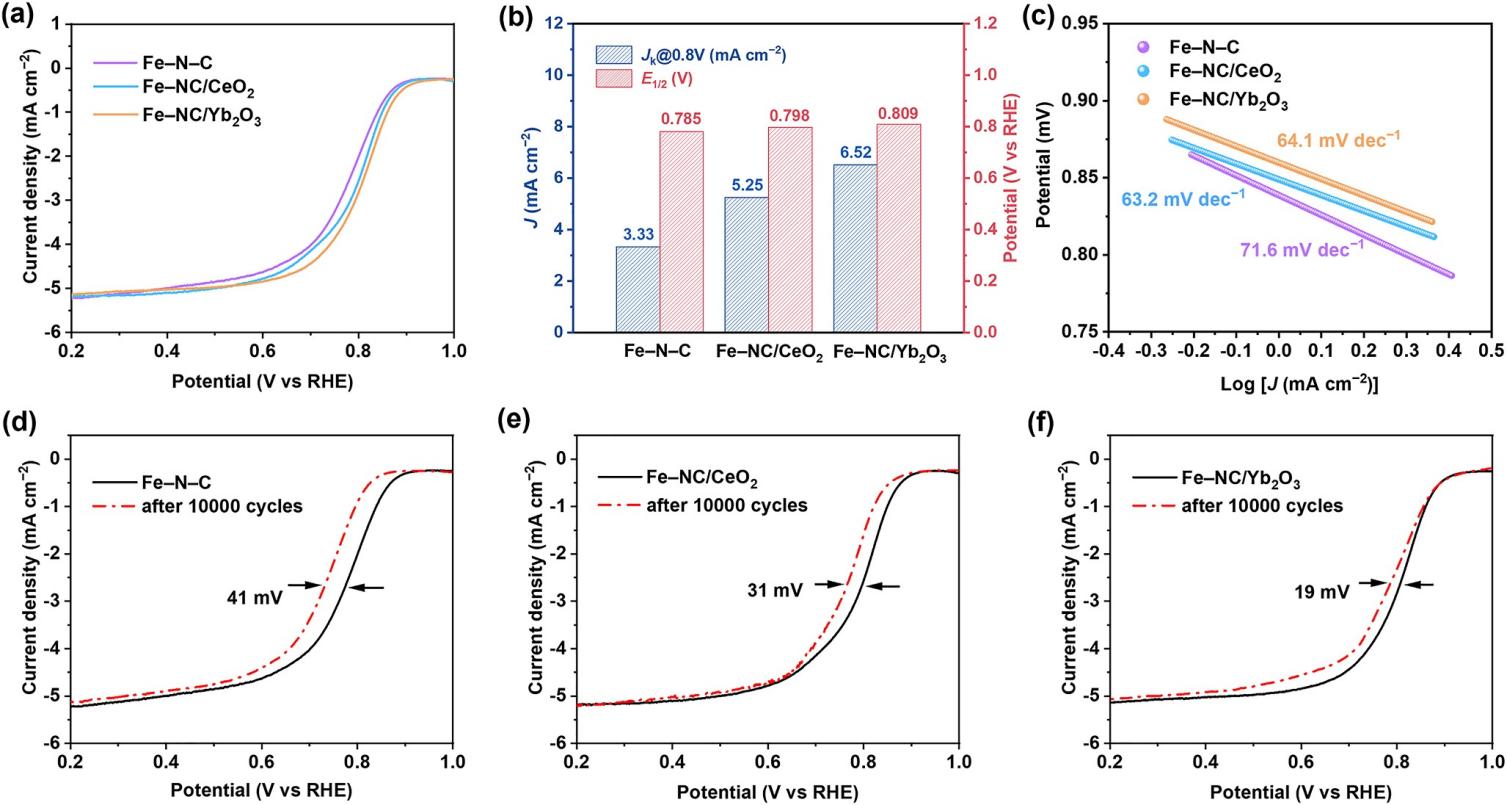
ORR activity and durability. (a) ORR polarization curves of Fe–N–C, Fe–NC/CeO_2_, and Fe–NC/Yb_2_O_3_. (b) Comparison of kinetic current density and half‐wave potential for Fe–N–C, Fe–NC/CeO_2_, and Fe–NC/Yb_2_O_3_. (c) Tafel slope comparison for Fe–N–C, Fe–NC/CeO_2_, and Fe–NC/Yb_2_O_3_. (d‐f) ORR polarization curves for Fe–N–C, Fe–NC/CeO_2_, and Fe–NC/Yb_2_O_3_ before and after 10,000 potential cycles in 0.5 м H_2_SO_4_. ORR, Oxygen reduction reaction.

To probe the durability enhancement resulting from the introduction of Yb_2_O_3_ scavengers near the Fe–N_4_ sites, accelerated stress tests were performed by potential cycling between 0.6 and 1.0 V for 10,000 cycles in O_2_‐saturated 0.5 м H_2_SO_4_ (Figure 3d–f). After cycling, Fe–NC/Yb_2_O_3_ showed the smallest *E*_1/2_ loss after cycling (only 19 mV), significantly outperforming Fe–N–C (41 mV) and Fe–NC/CeO_2_ (31 mV). Long‐term stability was further examined by chronoamperometry at 0.7 V (Figure S11). After 10 h, Fe–NC/Yb_2_O_3_ retained 85.6% of its initial ORR current, which was superior to the retentions of Fe–N–C (65.2%) and Fe–NC/CeO_2_ (69.3%). These results demonstrate that Yb_2_O_3_ nanoparticles possess the strongest radical‐scavenging capability, endowing Fe–NC/Yb_2_O_3_ with the highest catalytic stability.

To assess the stability of the radical scavengers, the concentrations of dissolved Yb and Ce in the electrolyte were measured after 10,000 potential cycles. A total catalyst loading of 1 mg was deposited onto the glassy carbon electrode to ensure detectable metal ion concentrations by inductively coupled plasma mass spectrometry. The results revealed a Yb concentration of 19.3 μg L^−1^ in the electrolyte (100 mL), corresponding to a loss of approximately 1.9% of the initial Yb_2_O_3_ loading. In comparison, the Ce concentration was 41.1 μg L^−1^, corresponding to a loss of about 5.2% of the CeO_2_. These findings indicate the superior chemical stability of Yb_2_O_3_ compared with traditional CeO_2_ under the operating conditions.

The practical performance of Fe–NC/Yb_2_O_3_ was further evaluated in a single‐cell PEMFC under 1 bar H_2_–air conditions. As shown in Figure 4a, the Fe–NC/Yb_2_O_3_ cathode delivered a peak power density (*P*_max_) of 0.32 W cm^−2^, outperforming both Fe–N–C and Fe–NC/CeO_2_ under the same conditions. Furthermore, during a 20 h stability test at a constant voltage of 0.5 V, Fe–NC/Yb_2_O_3_ retained 87.1% of its initial current density, representing a marked improvement over the 46.8% retention of Fe–N–C (tested for 13 h) and also exceeding the 74.9% retention of Fe–NC/CeO_2_ (Figure 4b). These results confirm the significantly enhanced operational stability of Fe–NC/Yb_2_O_3_ under realistic fuel cell conditions.

**FIGURE 4:**
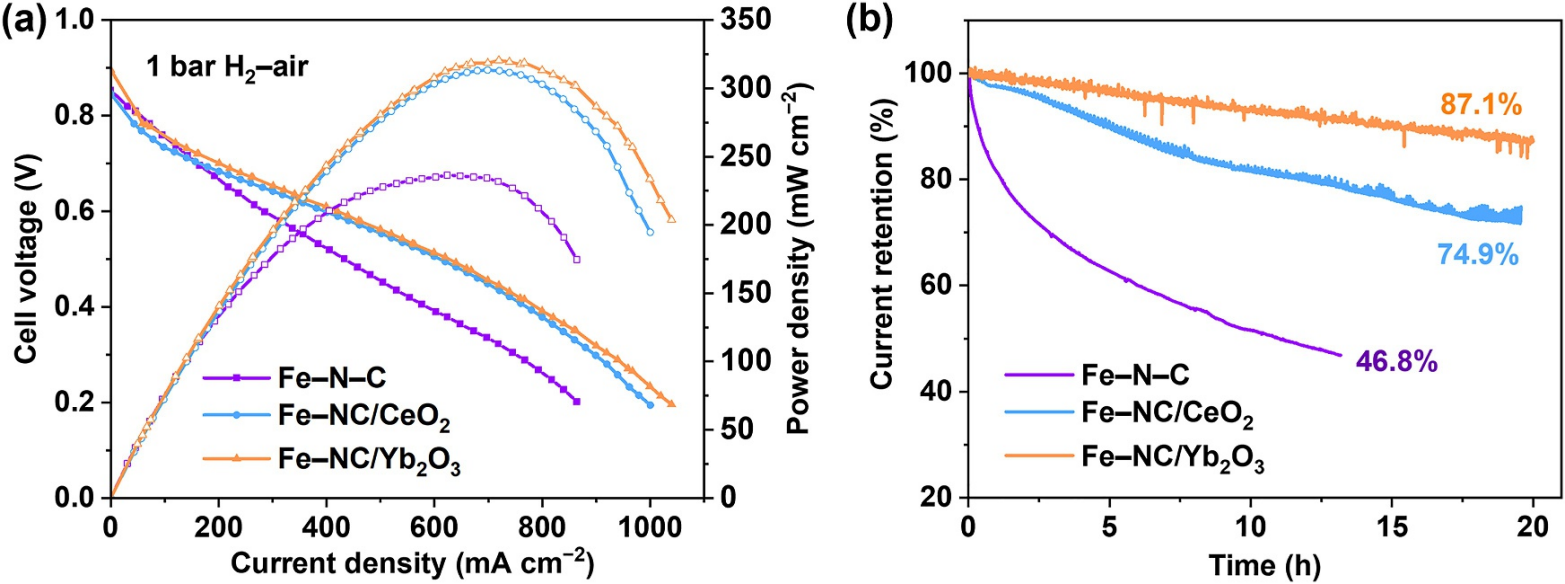
PEMFC performance. (a) Polarization and power density curves of H_2_–air PEMFCs with Fe–N–C, Fe–NC/CeO_2_, and Fe–NC/Yb_2_O_3_. (b) A 20‐h stability test for Fe–N–C, Fe–NC/CeO_2_, and Fe–NC/Yb_2_O_3_. PEMFC, Proton exchange membrane fuel cell.

### 2.4 Theoretical insights into the radical scavenging and activity enhancement mechanism

Density functional theory calculations were performed to elucidate how Yb_2_O_3_ enhances both the activity and durability of Fe–N_4_ sites. Models of the Fe–N_4_ site and the FeN_4_/Yb_2_O_3_ interface (Figure S12) were constructed. The charge density difference plot (Figure 5a) reveals a pronounced charge redistribution at the interface, indicating strong electronic coupling between Yb_2_O_3_ and the Fe–N_4_ moiety. This coupling modulates the electronic structure of the Fe center, as evidenced by a downshift of its *d*‐band center (*ε*_*d*) from −1.16 eV to −1.698 eV (Figure 5b). The lowered *ε*_*d* weakens the binding of oxygenated intermediates on Fe.

**FIGURE 5:**
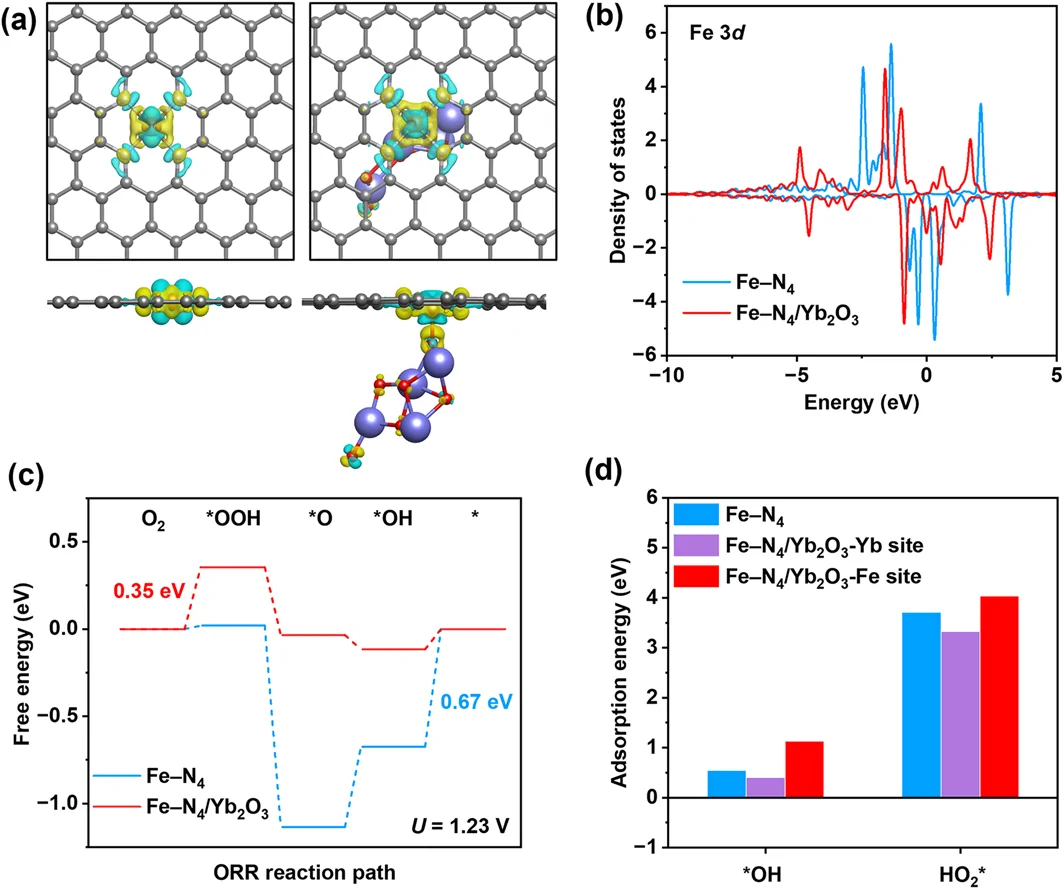
DFT analysis of the synergistic mechanism. (a) Charge density difference at the Fe–N_4_ and Fe–N_4_/Yb_2_O_3_ interfaces (isosurface = 0.009 e Å^−3^). Yellow and cyan denote electron accumulation and depletion, respectively. (b) Projected density of states (PDOS) of Fe 3d orbitals. (c) Calculated energy profiles for the oxygen reduction reaction (ORR) potential‐determining steps. (d) Adsorption energies of ·OH and HO_2_· radicals on relevant surfaces. Atom colors: Fe (orange), N (blue), C (gray), Yb (purple), O (red), H (white). DFT, Density functional theory.

This modification significantly alters the thermodynamic landscape of the ORR. For pristine Fe–N_4_, the strong Fe–OH interaction makes *OH desorption the potential‐determining step (PDS), requiring a free energy increase of 0.67 eV (Figure 5c). In the Yb_2_O_3_‐modified system, the weakened *OH binding shifts the PDS to *OOH formation, which requires only 0.35 eV. This value is also lower than that for a CeO_2_‐modified counterpart (0.47 eV, Figure S13) explaining the superior activity.

Yb_2_O_3_ also functions as a powerful radical scavenger. As shown in Figure 5d, the Yb sites in the composite strongly adsorb *OH and HO_2_* radicals, while adsorption on the modulated Fe–N_4_ site is weakened. This indicates the oxophilic nature of Yb_2_O_3_, which favors radical capture. Consequently, a protective mechanism is established wherein Yb_2_O_3_ competitively scavenges radicals, diverting them from the Fe–N_4_ sites. Since the Fe–N bond is susceptible to radical attack, this scavenging action mitigates Fe dissolution and carbon corrosion, thereby enhancing durability.

In summary, the density functional theory calculations reveal that Yb_2_O_3_ adjacent to the Fe–N_4_ sites provides a dual function: it tunes the Fe center to lower the ORR overpotential and concurrently acts as a radical scavenger to protect the active site. This mechanism jointly accounts for the experimentally observed synchronous enhancement in both activity and stability of the Yb_2_O_3_‐modified catalyst.

## 3 CONCLUSION

In summary, we developed a Fe–NC/Yb_2_O_3_ composite catalyst by immobilizing Yb_2_O_3_ nanoparticles near the Fe–N_4_ active sites, achieving instantaneous scavenging of radicals (·OH/·OOH) generated during the ORR. The high oxophilicity of Yb_2_O_3_ enables in situ radical capture, effectively suppressing radical migration and the consequent degradation of active sites. Yb_2_O_3_ also improves the electronic structure of Fe–N_4_ sites, contributing to the improved activity. As a result, the catalyst exhibits remarkable ORR activity, with an *E*_1/2_ of 0.809 V, and significantly enhanced stability, showing only a 19 mV loss in *E*_1/2_ after 10,000 cycles, which corresponds to a 40% improvement over the CeO_2_‐based system. In practical H_2_–air fuel cell tests, it retained 87.1% of its initial performance at 0.5 V, outperforming the CeO_2_‐modified catalyst (74.9%). This work reports Yb_2_O_3_ as a radical scavenger in fuel cells, offering a viable strategy to overcome the longstanding activity–stability trade‐off in non‐precious metal catalysts.

## CONFLICT OF INTEREST STATEMENT

The authors declare no conflicts of interest.

## ETHICS STATEMENT

No animal or human experiments were involved in this study.

## Supporting information

Supporting Information S1

## Data Availability

The data that support the findings of this study are available from the corresponding author upon reasonable request.
