# Supplementary material for: Oxophilic Yb2O3 nanoparticles as radical scavengers to enhance the durability of Fe–N–C oxygen reduction catalysts
Source: Smart Mol. 2026 May 6:e70049. Online ahead of print. doi: 10.1002/smo2.70049 (PMC13398919; doi:10.1002/smo2.70049)
Supplement: Supplementary file 1 — Supporting Information S1 [file SMO2-9999-0-s001.docx]

Supporting Information

**Oxophilic Yb_2_O_3_ nanoparticles as radical scavengers to enhance the durability of Fe‒N‒C oxygen reduction catalysts**

*Jiayu Liu, Shuang Li, Huijuan Li, Xin Wan*, and Jianglan Shui**

J. Liu, S. Li, H. Li, X. Wan, J. Shui

School of Materials Science and Engineering, Beihang University, Beijing, 100191, China
E-mail: wanxin@buaa.edu.cn; shuijianglan@buaa.edu.cn

J. Shui
Tianmushan Laboratory, Hangzhou, 311115, China

**Experimental Section**

**Synthesis of ZIF-8.** A methanol solution (50 mL) containing 2.94 g of Zn(NO_3_)_2_·6H_2_O was added to another methanol solution (100 mL) containing 3.24 g of 2-methylimidazole. The mixture was magnetically stirred at room temperature for 12 hours. The product was collected by centrifugation, washed with methanol three times, and dried overnight under vacuum at 80°C.

**Synthesis of NC.** 1.0 g of the obtained ZIF-8 and 0.3 g of 1,10-phenanthroline were dispersed in a mixed solvent of ethanol and deionized water (2:1 v/v). The mixture was magnetically stirred at room temperature for 12 hours. After drying overnight at 80°C under vacuum, the product was ground into a fine powder and then maintained at 900°C for 2 hours under a continuous argon flow. Subsequently, the sample was allowed to cool naturally to room temperature.

**Synthesis of NC/Yb_2_O_3_.** 60 mg of NC and 10, 15, or 20 mg of Yb(NO_3_)_3_·5H_2_O were dispersed in 20 mL of ethanol. After ultrasonication for 30 min, the mixture was stirred and evaporated in a water bath at 50°C. The resulting powder was then heated to 120°C under argon for low-temperature annealing. Subsequently, the temperature was further increased to 900°C, and pyrolysis was carried out at this temperature for 2 hours. After natural cooling to room temperature, the final product NC/Yb_2_O_3_ was obtained.

**Synthesis of Fe–NC/Yb_2_O_3_.** 8 mg of ferrocene and 50 mg of NC/Yb_2_O_3_ were thoroughly ground together and then subjected to annealing. The mixture was heated to 120°C under argon for low-temperature annealing. Subsequently, the temperature was further increased to 900°C for pyrolysis, followed by natural cooling to room temperature.

**Synthesis of Fe–NC/CeO_2_.** NC/CeO_2_ composite was synthesized following the same procedure used for NC/Yb_2_O_3_, except that Yb(NO_3_)_3_·5H_2_O was replaced with an equal mass of Ce(NO_3_)_3_·6H_2_O. The subsequent iron function procedure was identical to the synthesis of Fe–NC/Yb_2_O_3_.

**Characterization.** A JEOL JEM-2100F microscope (operating voltage 200 kV) was used to observe the micro-morphology and structure of the materials. Samples were prepared by ultrasonically dispersing the powder in isopropanol. After standing, the upper suspension was dropped onto an ultrathin carbon support film or a microgrid and dried under vacuum before observation. Phase analysis was performed using a Rigaku D/MAX-2500 diffractometer. Test conditions: Cu Kα radiation source, operating voltage 40 kV, current 200 mA; scanning range 5–80°, step size 0.02°, scanning rate 6° min^−1^. Sample preparation: For fine powders, they were compacted into a grooved glass slide; for samples with larger particles or poor adhesion, they were leveled and fixed by dripping anhydrous ethanol. A Thermo ESCALAB 250Xi spectrometer (Al Kα excitation source) was used to analyze the surface elemental composition, chemical states, and relative content of the materials. Data were processed using XPSPEAK software, with background subtraction via the Shirley method and least-squares fitting. The Yb or Ce concentration measurements were conducted on the Agilent 5110 ICP-OES or 7850 ICP-MS.

**Electrochemical Measurements.** All electrochemical measurements were performed on a CHI 760e electrochemical workstation using a standard three-electrode system. A saturated calomel electrode (SCE) was used as the reference electrode, and a graphite rod served as the counter electrode. A glassy carbon (GC) electrode was used as the working electrode. 215 µL of Nafion solution (5 wt%, Aldrich), 4.3 mL of deionized water, and 5.485 mL of isopropanol were mixed to obtain a Nafion solution with a concentration of 1 mg mL^−1^. 1 mg of catalyst powder was weighed and dispersed in 100 µL of the above Nafion solution. The mixture was ultrasonicated for 30 minutes to obtain a homogeneous suspension. The surface of the glassy carbon electrode for the rotating disk electrode (RDE) or rotating ring-disk electrode (RRDE) was sequentially polished with 0.3 µm and 50 nm alumina powder and thoroughly rinsed with deionized water. Then, 10 µL of the catalyst ink was pipetted and dropped onto the glassy carbon surface of the electrode (diameter 5.61 mm) to ensure uniform coverage of the entire disk area. The electrode was dried at room temperature or under a gentle air flow to form a uniform catalyst layer.

RRDE measurements were conducted via linear sweep voltammetry (LSV) in a potential range of −0.1 V to 0.8 V at a rotation speed of 1600 rpm and a scan rate of 10 mV s^−1^. The electron transfer number (*n*) and hydrogen peroxide yield (H_2_O_2_%) were calculated using the following formulas:

H_2_O_2_(%) = 200 × (*I*_r_ / *N*) / (*I*_r_ / *N* + *I*_d_) (1)

*n* = 4 *I*_d_ / (*I*_r_ / *N* + *I*_d_) (2)

where *I*_d_ is the disk current, *I*ᵣ is the ring current, and *N* (0.37) is the current collection efficiency of the Pt ring. The kinetic current density (*j*ₖ) involved in the ORR process was determined by analyzing the Koutecky-Levich (K-L) equation:

1/*j* = 1/*j*_k_ + 1/*j*_L_ (3)

where *j* is the measured current density and *j*_L_ is the diffusion-limited current density.

Accelerated durability tests (ADT) were performed in O_2_-saturated 0.5 M H_2_SO_4_ solution at 25 °C by applying cyclic potential scans between 0.6 V and 1.0 V (vs. RHE) at a scan rate of 50 mV s^−1^ for 10,000 cycles.

**PEMFC Test.** 15 mg of Fe-based catalyst was mixed with 200 mg of deionized water, 400 mg of isopropanol, and 210 mg of Nafion solution (5 wt%). The mixture was ultrasonicated for 30 minutes and stirred for 12 hours to form a homogeneous suspension. Pt/C anode ink was prepared by mixing 2 mg of 40 wt% Pt/C catalyst with 16.8 mg of Nafion solution, deionized water, and isopropanol (mass ratio 1:2), followed by 30 minutes of ultrasonication and 2 hours of stirring. The membrane electrode assembly (MEA) was formed by coating the catalyst onto a 5 cm^2^ carbon paper (GDS2240), vacuum-drying at 80°C for 4 hours, and then hot-pressing with an NR211 membrane to form a sandwich structure. Performance testing was conducted on a Scribner 850e test station under conditions of 80°C and 100% relative humidity, with H_2_/air flow rates of 0.3/0.5 L·min⁻¹ and pressures of 1 bar. Polarization curves were scanned in steps of 20 mA·cm^−2^ with a 3 s equilibrium time. The fuel cell test temperature was 80°C.

**Radical Scavenging Assay.** This study employed the ABTS [2,2‑azinobis(3‑ethylbenzothiazoline‑6‑sulfonate)] radical scavenging assay to evaluate the radical scavenging activity of three catalysts: Fe–N–C, Fe–NC/CeO_2_, and Fe–NC/Yb_2_O_3_. The experimental procedure is as follows: Each catalyst (4.0 mg) was separately ultrasonically dispersed in 5 mL of 0.1 M H_2_SO_4_ solution to form a homogeneous suspension. Appropriate amounts of ABTS and H_2_O_2_ were sequentially added to achieve final concentrations of 2 mM and 10 mM, respectively. The mixture was allowed to react at room temperature for 10 minutes. After 10 minutes, the solution was centrifuged, and the supernatant was diluted 1:50 with 0.1 M H_2_SO_4_ solution. 2 mL of the diluted ABTS·⁺ solution was placed in a test container. The absorbance of the reaction system was measured using a UV-Vis spectrophotometer. The radical scavenging ability of the catalysts was evaluated by monitoring the absorbance change at 417 nm. A greater decrease in absorbance indicates stronger radical scavenging activity of the catalyst.

**Computational Methods.** The oxygen reduction reaction (ORR) mechanism on the catalysts was simulated using computational methods based on density functional theory (DFT) with the Vienna Ab Initio Simulation Package (VASP). In the calculations, the interaction between valence electrons and ionic cores was treated using the Projector Augmented Wave (PAW) potential, and the Perdew-Burke-Ernzerhof (PBE) form of the Generalized Gradient Approximation (GGA) functional was employed to describe electron exchange-correlation energy. After a series of model tests, the plane-wave cutoff energy was set to 500 eV. Brillouin zone sampling used the Monkhorst-Pack method with a k-point grid of 3×3×1. The convergence criterion for ionic relaxation steps was set to an energy change less than 0.01 eV Å^−1^. To eliminate interactions between adjacent surfaces under periodic boundary conditions, a vacuum layer of 25 Å was set in the z-direction of the model.

The four-electron pathway for ORR proceeds according to the following elementary steps:

* + O_2_(g) + H^+^ + *e*^−^ → OOH*

OOH* + H^+^ + *e*^−^ → O*

O* + H^+^ + *e*^−^ → OH*

OH* + H^+^ + *e*^−^ → * + H_2_O(l)

In addition to the above steps, the two-electron pathway for hydrogen peroxide formation can occur due to relative free energies:

* + O_2_(g) + H^+^ + *e*^−^ → OOH*

OOH* + H^+^ + *e*^−^ → * + H_2_O_2_(l)

Here, * represents the active site, and OOH*, O*, and OH* refer to adsorbed intermediates.

**Supplementary Figures**


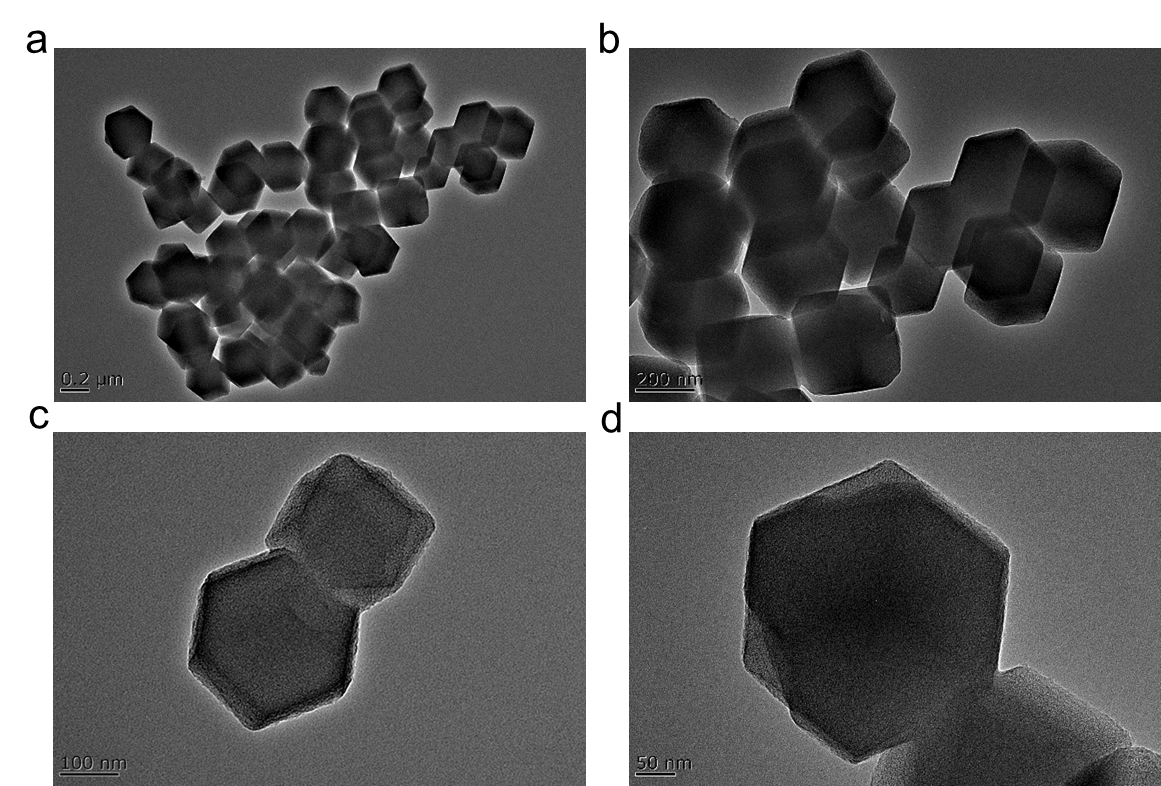


**Figure S1.** (a-d) TEM images of NC.


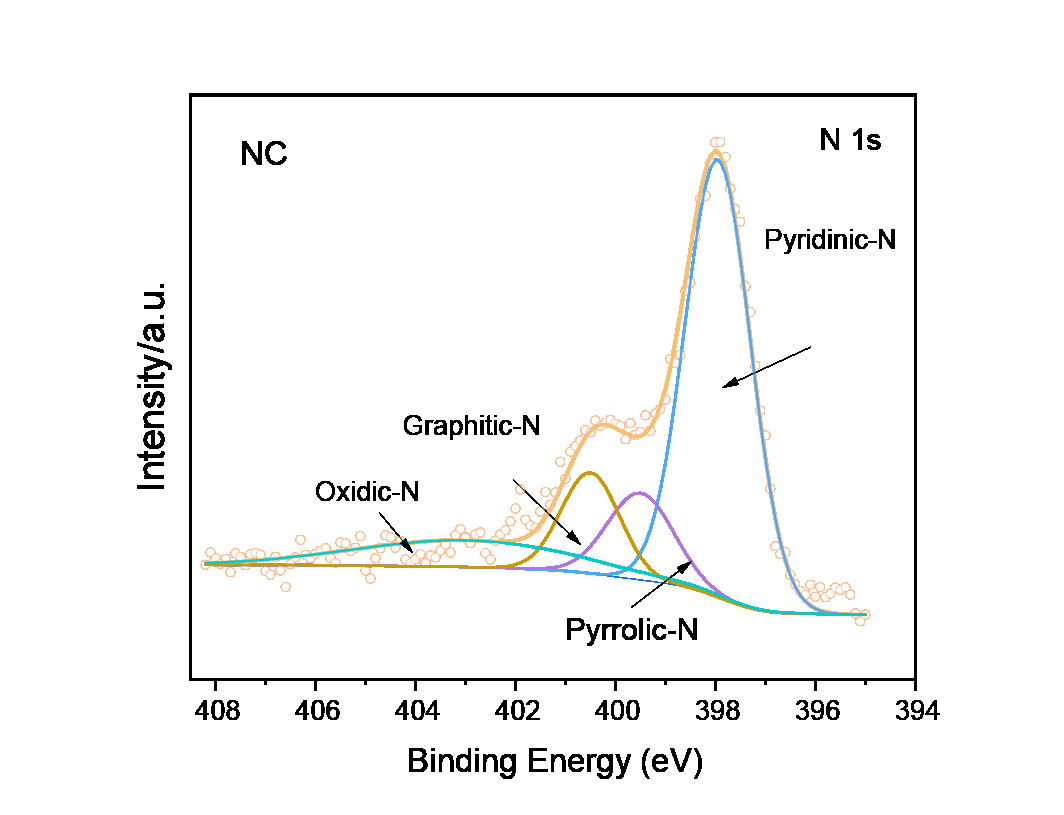


**Figure S2.** XPS N 1s spectrum of NC.


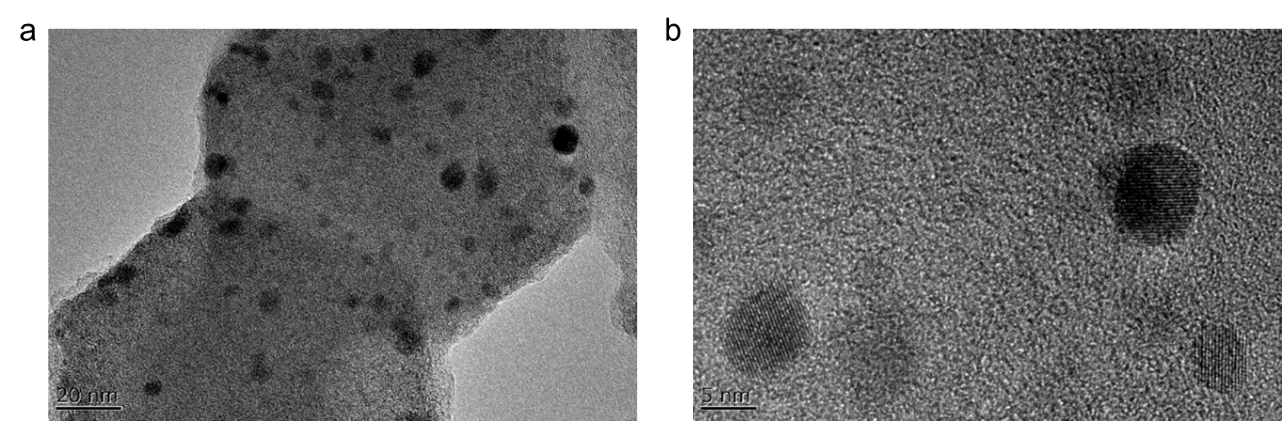


**Figure S3.** TEM images of NC/Yb_2_O_3_.


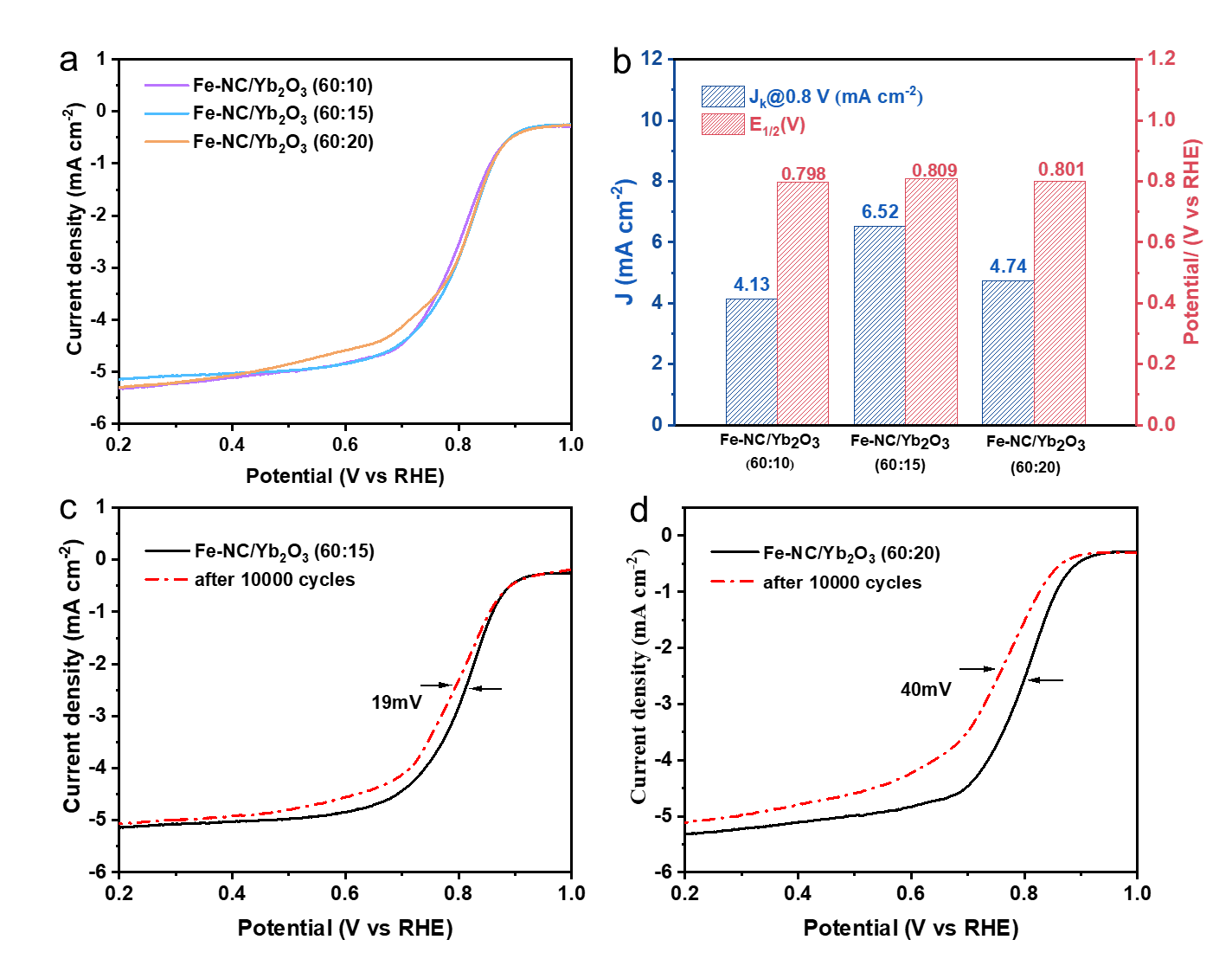


**Figure S4.** a) ORR polarization curves of Fe–NC/Yb_2_O_3_(60:10), Fe–NC/Yb_2_O_3_(60:15), and Fe–NC/Yb_2_O_3_(60:20). b) ORR polarization curves of Fe–NC/Yb_2_O_3_(60:15) before and after 10,000 potential cycles in 0.5 M H_2_SO_4_. c) ORR polarization curves of Fe–NC/Yb_2_O_3_(60:20) before and after 10,000 potential cycles in 0.5 M H_2_SO_4_.


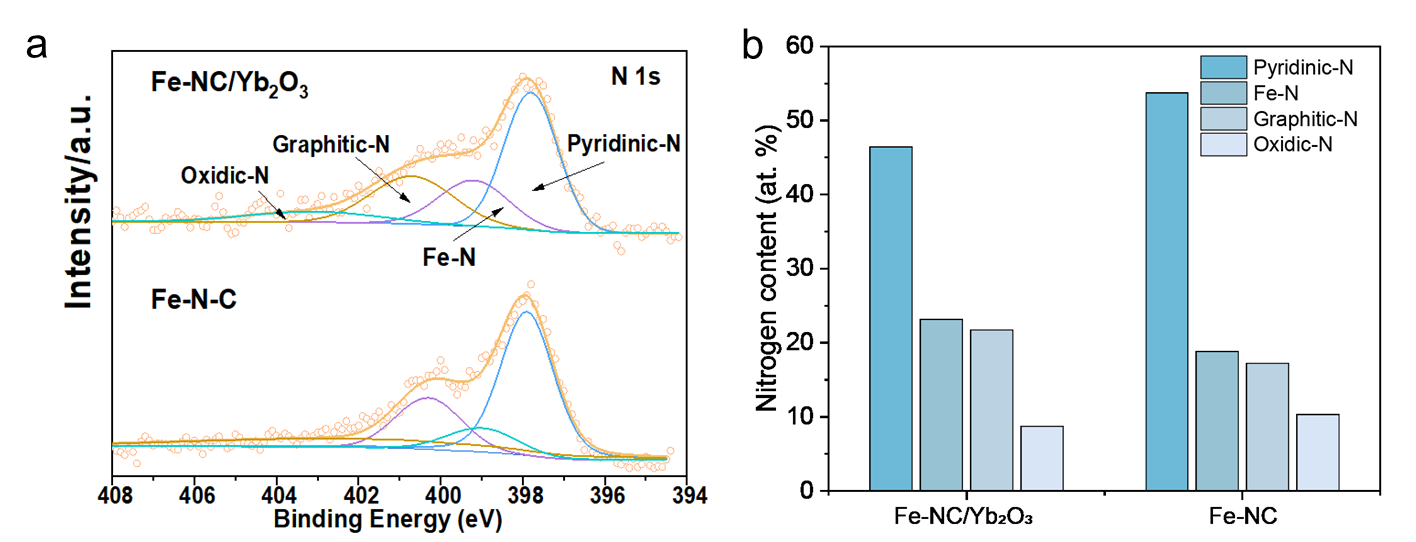


**Figure S5.** (a) XPS N1s spectra of Fe–N–C and Fe–NC/Yb_2_O_3_ and (b) fractions of various nitrogen species.


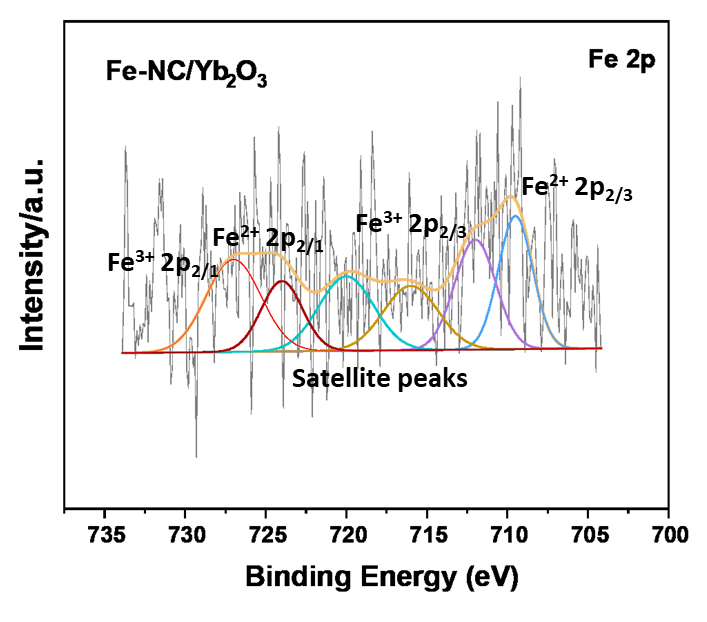


**Figure S6.** XPS Fe 2p spectra of Fe–NC/Yb_2_O_3_.


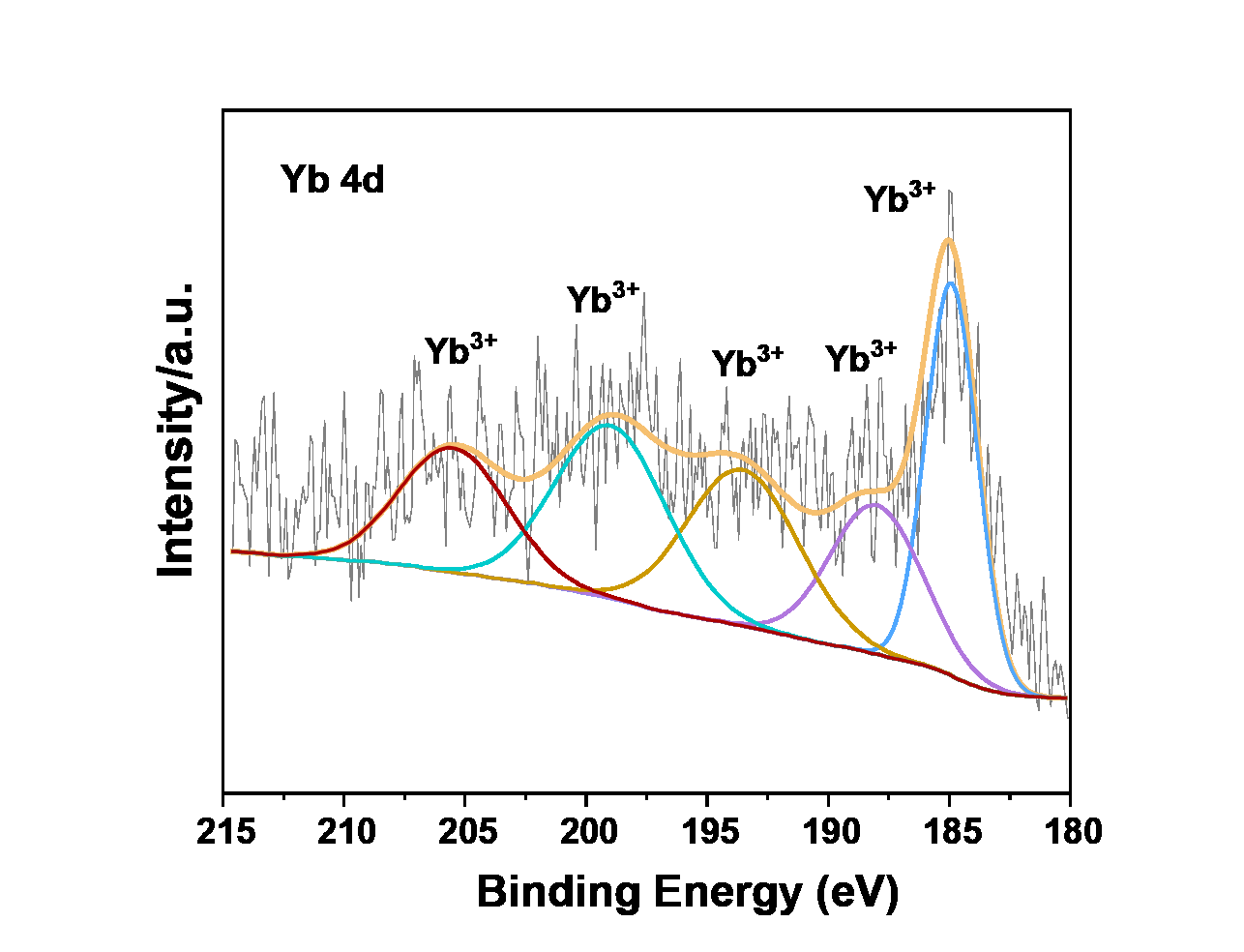


**Figure S7.** XPS Yb 4*d* spectrum of Fe–NC/Yb_2_O_3_.


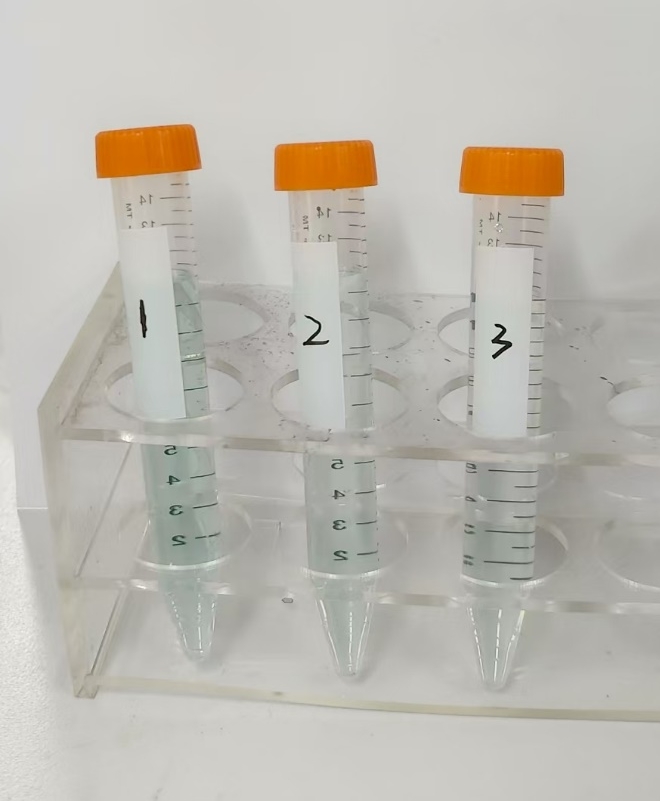


**Figure S8.** Color change of the ABTS solution after reaction with radicals generated by the Fenton reaction under different catalysts (1. Fe–N–C, 2.Fe–NC/CeO_2_, and 3. Fe–NC/Yb_2_O_3_).


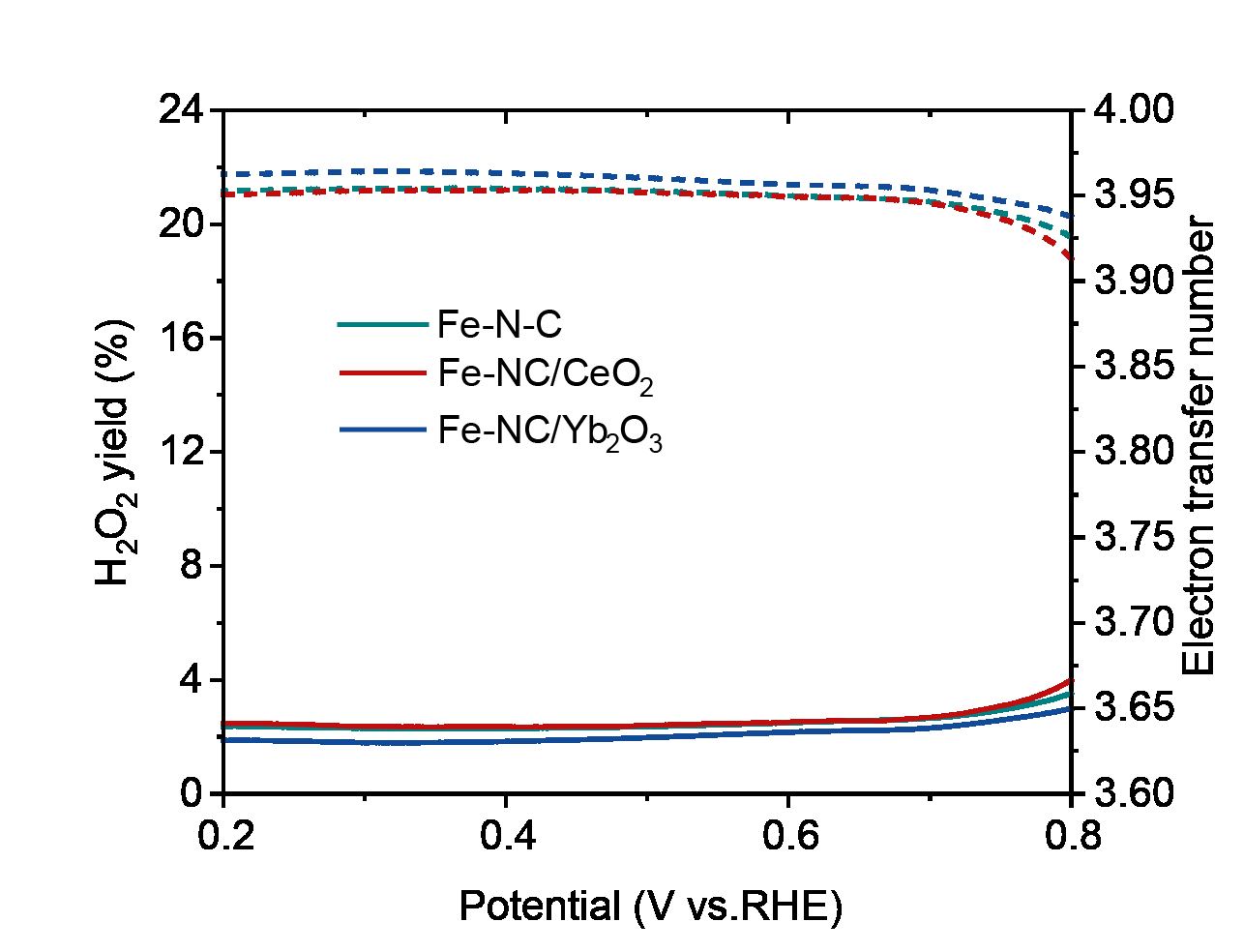


**Figure S9.** Electron transfer number (*n*) (top) and H_2_O_2_ yield (bottom) for Fe–N–C, Fe–NC/CeO_2_, and Fe–NC/Yb_2_O_3_.


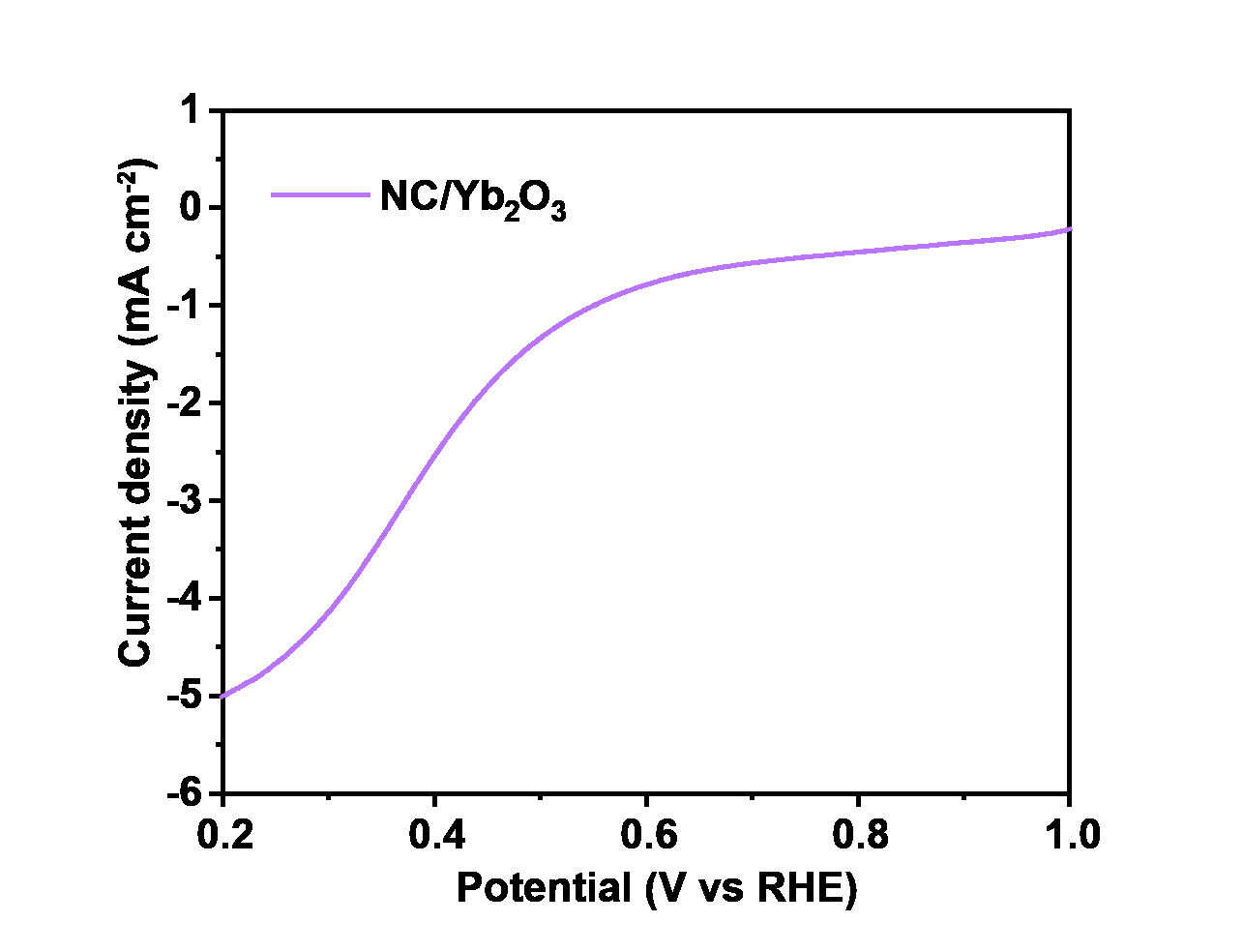


**Figure S10.** ORR polarization curves of NC/Yb_2_O_3_.


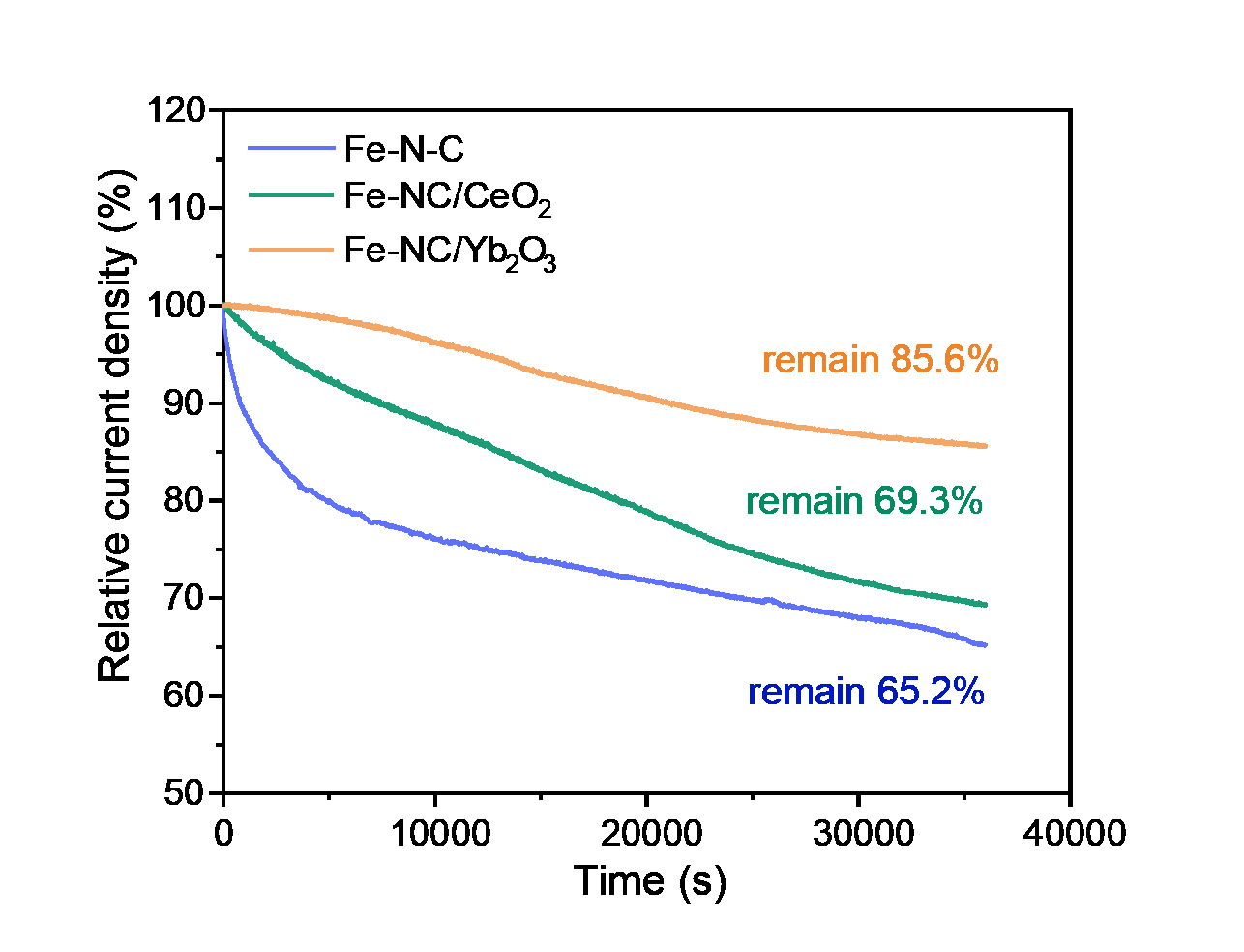


**Figure S11.** A 10-h stability test of Fe–N–C, Fe–NC/CeO_2_, and Fe–NC/Yb_2_O_3_ catalysts at a constant potential of 0.7 V.


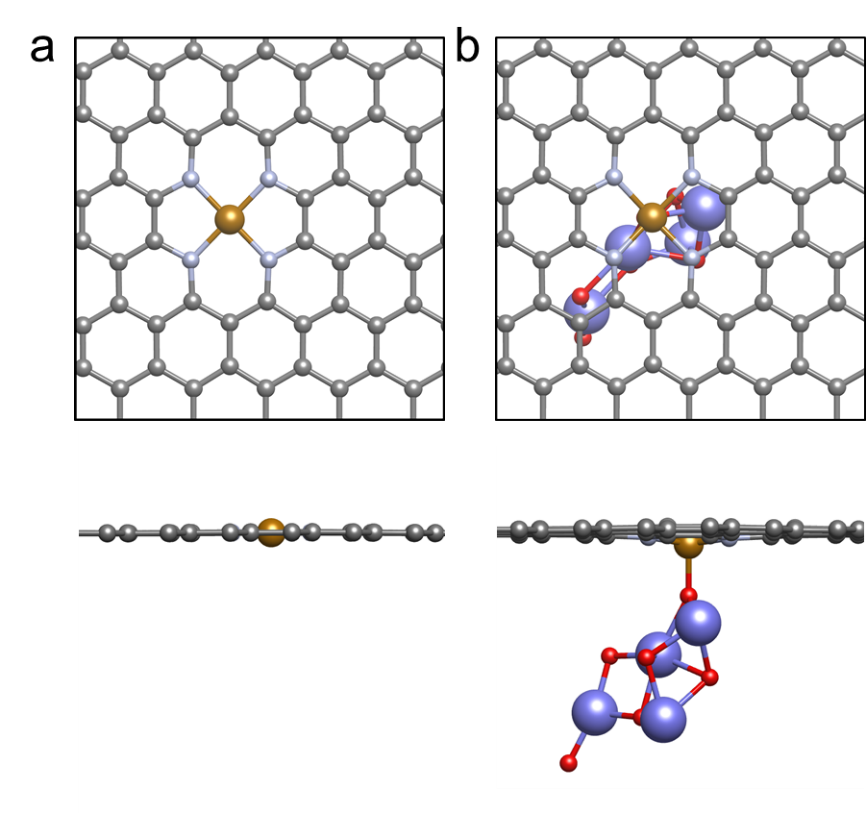


**Figure S12.** (a) Side and top views of the pristine Fe–N_4_ site model embedded in a graphene layer. (b) Side and top views of the Fe–N_4_/Yb_2_O_3_ structural model. Color code: Fe (orange), N (blue), C (gray), Yb (purple), O (red).


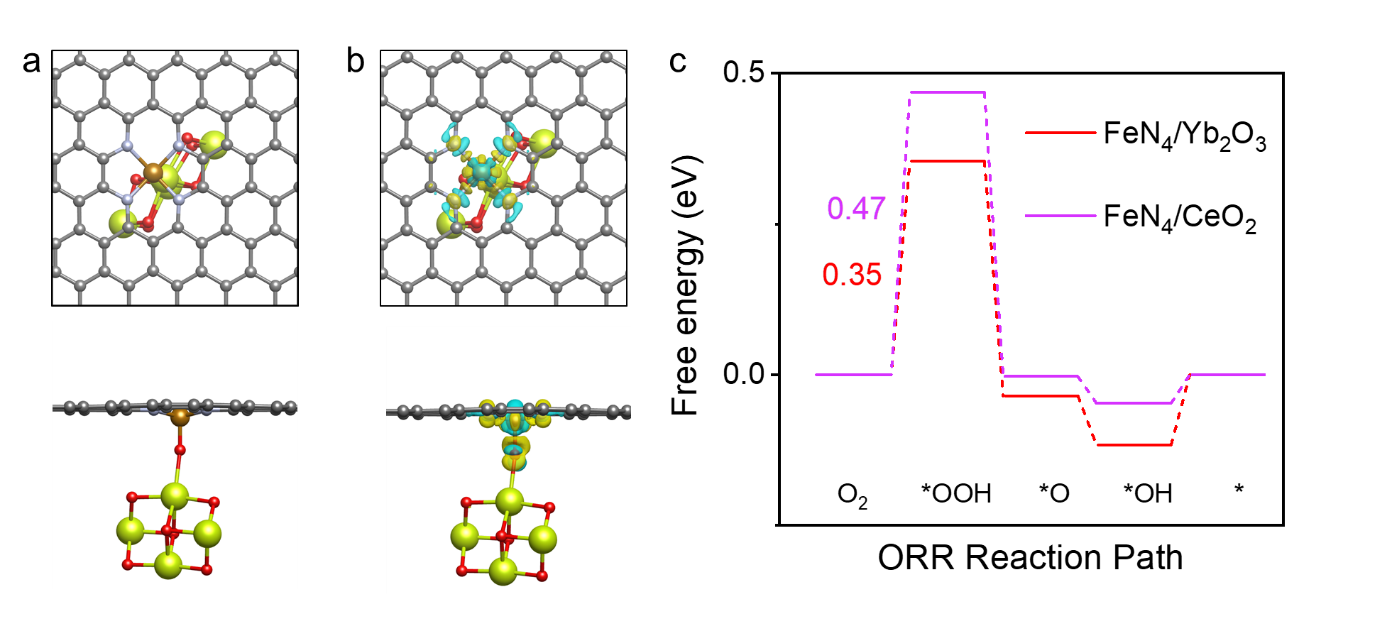


**Figure S13.** DFT analysis of the CeO_2_-modified system. (a) Atomic structure model of the Fe–N_4_/CeO_2_ interface. (b) Charge density difference at the interface (isosurface = 0.009 e Å^−3^). Yellow and cyan denote electron accumulation and depletion, respectively. (c) Calculated energy profiles for the ORR on the Fe–N_4_/CeO_2_ and Fe–N_4_/Yb_2_O_3_ catalysts. Atom colors: Fe (orange), N (blue), C (gray), Ce (golden), O (red), H (white).
